# Macro-AST: misleading finding in an adolescent with MCAD-deficiency

**DOI:** 10.1186/1471-230X-12-119

**Published:** 2012-08-30

**Authors:** Anibh M Das, Sabine Drache, Nils Janzen, Andreas Franke

**Affiliations:** 1 Department of Paediatrics, Hannover Medical School, Carl-Neuberg Str. 1, D-30625, Hannover, Germany; 2 MVZ Labor Dr. Eberhard & Partner, Dortmund, Germany; 3 Department of Internal Medicine II, Siloah Hospital, Hannover, Germany; 4 Present address: Department of Neuropaediatrics, Ruhr University, Bochum, Germany

**Keywords:** Macro-AST, MCAD-deficiency, Liver, Heart, Muscle, Immunoglobulin

## Abstract

**Background:**

MCAD-deficiency is the most common inborn error of fatty acid oxidation now included in many newborn screening programms using MS/MS. During prolonged catabolic episodes, patients may suffer from metabolic decompensation with dysfunction of liver, skeletal- and heart muscle as well as brain. In anabolism, neither clinical symptoms nor biochemical signs of organ dysfunction occur.

**Case presentation:**

We report a female patient with MCAD-deficiency in whom at the age of 11 years isolated AST-elevation was found without any clinical or biochemical signs of organ dysfunction. We showed by polyethylene glycol precipitation that macro-AST formation was responsible for this biochemical finding. AST was probably complexed with immunoglobulins possibly related to an allergic disposition. Macro-AST formation is not a special feature of MCAD-deficiency but rather a non-specific, coincidental finding which also occurs in healthy individuals. The general practitioner consulted by the patient before coming to our outpatient clinic for inborn errors of metabolism was worried that isolated AST-elevation indicated cell damage in MCAD-deficiency. He ordered further diagnostic tests like ultrasound, ECG and echocardiography without any pathology.

**Conclusion:**

In isolated AST-elevation, macro-AST has to be considered in order to avoid unnecessary, costly and invasive evaluation. This is not only true for healthy persons but for patients with chronic diseases like MCAD as well.

## Background

Medium chain acyl CoA-dehydrogenase (MCAD)-deficiency (OMIM #201450) is the most common inborn error of mitochondrial fatty acid oxidation. It is inherited as an autosomal-recessive trait and leads to compromised breakdown of C4-C12 acyl-CoAs. The incidence in Northern Europe is 1:9,000 to 1:11,000 ( [1] and own data). Catabolism (immediately after birth or during common infections) may trigger acute metabolic decompensation. As fatty acids are the main energy fuel for skeletal and heart muscle as well as liver, dysfunction of these organs may occur during metabolic decompensation in MCAD-deficient patients though other mechanisms may play an additional role. Encephalopathy is presumably caused by the accumulation of toxic lipophilic compounds proximal to the enzyme defect as well as hypoketotic hypoglycaemia. Hypoketonaemia is based on compromised hepatic ketogenesis resulting from reduced fatty acid oxidation and leads to energy deprivation of the brain during prolonged catabolism/fasting.

MCAD-deficiency is a target disease of many newborn screening programs using the MS/MS technique which resulted in a significant reduction of metabolic decompensations and death by simply avoiding catabolism. The same goes for MCAD-patients diagnosed after an initial metabolic decompensation by selective screening.

There is no chronic toxicity known in MCAD-deficiency though prospective long-term studies are lacking. In childhood, decompensation typically occurs during febrile illness or emesis. However, there are different challenges during adolescence/adulthood which may hamper anabolism in the absence of parental care [2]. Catabolism may be triggered by alcohol ingestion, fasting for weight reduction and competitive sports without adequate intake of food, in females, pregnancy and delivery may be an issue.

We report a female patient with MCAD-deficiency who developed isolated AST-elevation at the age of 11 years which was supposed to be a sign of chronic toxicity but later turned out to be due to macro-AST. Macro-AST is known for many years as a cause of isolated AST-elevation, however, it is often not considered during the work-up of patients with AST-elevation.

## Case presentation

The girl was born before the era of newborn screening for MCAD-deficiency by tandem MS/MS.

After an uneventful pregnancy and delivery at term she presented with 3 generalized tonic seizures associated with hypoglycaemia at the age of 3 days. Selective screening led to the diagnosis of MCAD-deficiency, homozygosity for the mutation p.K329E was found. Parents were advised to avoid catabolism, she did not have any further metabolic decompensation and showed normal psychomotor development. In the first years of life, she was regularly seen by a metabolic specialist, later –after the family moved out of town- she was seen by a general practitioner who sporadically took blood to assess organ functions. The girl developed allergic rhinitis and asthma, birch-specific IgE was elevated. At the age of 11 years, isolated AST-elevation (700 U/l, normal < 31 U/l) was first noticed. AST-levels remained elevated ever since (500–700 U/l) without any other laboratory abnormalities. Ultrasound of the abdomen, ECG and echocardiography were repeatedly normal, infection with hepatotropic viruses and autoimmune processes were excluded. The general practitioner concluded that isolated AST-elevation was related to MCAD-deficiency and indicates some organ dysfunction though the organ affected could not be specified yet.

The girl first presented at our outpatient clinic for inborn errors of metabolism at the age of 15 years. Psychomotor development was normal, physical examination was unrevealing. In line with MCAD-deficiency, the acylcarnitine profile in a dried blood spot showed elevations of C6, C8, C10 and C10:1 acylcarnitines, free carnitine concentration was normal. Analyses of amino acids in plasma and organic acids in urine were unrevealing. Concentrations of creatine kinase, ALT, alkaline phosphatase, gamma-glutamyltransferase, lipase and lactate dehydrogenase were not elevated there were no signs of hemolysis. Blood count was normal except for eosinophilia indicating an allergic disposition. AST was elevated (598 U/l, normal: < 31 U/l). In the absence of clinical and biochemical organ dysfunction, we considered macro-AST. More than 95% of AST-activity could be precipitated by polyethylene glycol indeed, thus confirming the presence of macro-AST. Macro-AST is supposed to result from complex formation with immunoglobulins which prompted us to determine immunoglobulin concentrations in blood. IgE-levels were above normal (412 IU/ml, reference range 1–100 IU/ml), probably related to birch allergy already diagnosed during childhood, while all other immunoglobulins were not elevated.

Informed consent for publishing this case was obtained from the parents and the patient.

## Conclusions

MCAD-deficiency can lead to organ damage (especially skeletal- and heart muscle, liver, brain) as a result of cellular energy deficiency and/or accumulation of endogenous toxic intermediates. Hypoketotic hypoglycaemia based (amongst other factors) on compromised hepatic ketogenesis from fatty acids is another pathophysiological mechanism leading to encephalopathy during prolonged fasting or intercurrent illness. Organ dysfunction is triggered by longer episodes of catabolism (febrile infection/prolonged fasting) and can usually be prevented by administering carbohydrates. Cell damage leads to spill-over of intracellular enzymes like AST, ALT, creatine kinase, lactic dehydrogenase into the blood circulation. Usually, this does not occur when the patient is well nor does it lead to isolated leakage of intracellular AST. However, metabolites accumulating in MCAD-deficiency are known to be toxic and may lead to organ dysfunction in the long run. There are no large prospective studies describing the outcome of patients diagnosed by neonatal mass screening in adulthood. Therefore, the assumption by the general practitioner that sustained, isolated AST-elevation indicated organ damage in our patient was reasonable. We could show that isolated AST-elevation in this MCAD-patient was caused by macro-AST. This was not a congenital condition as AST-levels were normal initially.

Macro-AST has been described in the literature as a benign condition due to binding of AST to macromolecules like immunoglobulins (e.g. [3-6]). These complexes cannot be excreted via the kidneys, therefore their half-life is extended. In our patient, we found elevated immunoglobulin E resulting from an allergic disposition while all other immunoglobulins were normal. In most cases described so far, AST was complexed with immunoglobulins A, M and G [7].

Macro-AST has not only been described in healthy persons but also in patients with hepatic diseases like hepatitis C [8] or malignancies and chronic liver disease [9]. Macro-AST or isolated AST-elevation is not a common feature in MCAD-deficiency. As Macro-AST is fairly common in the general population [7] macro-AST in our MCAD-patient is coincidental.

In summary, we describe a girl with MCAD-deficiency developing isolated AST-elevation without any clinical or biochemical signs for organ dysfunction. We could detect macro-AST as the cause of AST-elevation.

Macro-AST has to be considered in any healthy individual or patient with a chronic disease displaying an isolated AST-elevation. This goes for patients with chronic metabolic diseases like MCAD-deficiency as well. Thus, unnecessary costly and invasive evaluation can be avoided. In our opinion, MCAD-patients should be regularly followed. Apart from dealing with acute health problems this will allow us to study the long-term biochemical and clinical outcome in patients with this rare disease diagnosed by neonatal mass screening.

## Abbreviations

ALT: Alanine aminotransferase; AST: Aspartate aminotransferase; ECG: Electrocardiogram; MCAD: Medium chain acyl-CoA dehydrogenase; MS/MS: Tandem-mass spectrometry.

## Competing interests

The authors declare that they have no competing interests.

## Authors’ contributions

AMD treated the patient in the metabolic outpatient clinic, drafted and approved the manuscript. NJ and SD performed the analysis for macro-AST and advised regarding interpretation of results. NJ assisted in drafting the manuscript. AF performed ultrasound, ECG, echocardiography and various blood tests in the patient. All authors read and approved the final manuscript.

## Pre-publication history

The pre-publication history for this paper can be accessed here:

http://www.biomedcentral.com/1471-230X/12/119/prepub
